# Do we need a different organ allocation system for kidney transplants using donors after circulatory death?

**DOI:** 10.1186/1471-2369-15-83

**Published:** 2014-05-22

**Authors:** Shanka K Benaragama, Teressa Tymkewycz, Biku J John, Andrew Davenport, Ben Lindsey, David Nicol, Jonathon Olsburgh, Martin Drage, Nizam Mamode, Francis Calder, John Taylor, Geoff Koffman, Nicos Kessaris, Mohamed Morsy, Roberto Cacciola, Carmelo Puliatti, Susana Fernadez-Diaz, Asim Syed, Nadey Hakim, Vassilios Papalois, Bimbi S Fernando

**Affiliations:** 1 UCL Centre for Nephrology, Royal Free hospital, London, UK; 2 South Central Organ Donation Services, London, UK; 3 Department of Renal Transplantation, Guys and St. Thomas’ Hospital, London, UK; 4 Department of Renal Transplantation, St George’s Hospital, London, UK; 5 Department of Renal Transplantation, Royal London & St Bart’s NHS Trust, London, UK; 6 West London Renal Transplant Centre, Hammersmith Hospital, London, UK; 7 Centre for Nephrology and Transplantation, Royal Free London NHS Trust, Pond Street, London NW3 2QG, UK

**Keywords:** Donation after circulatory death, Allocation, Kidney, Delayed graft function, Cold ischemia time

## Abstract

**Background:**

There is no national policy for allocation of kidneys from Donation after circulatory death (DCD) donors in the UK. Allocation is geographical and based on individual/regional centre policies. We have evaluated the short term outcomes of paired kidneys from DCD donors subject to this allocation policy.

**Methods:**

Retrospective analysis of paired renal transplants from DCD’s from 2002 to 2010 in London. Cold ischemia time (CIT), recipient risk factors, delayed graft function (DGF), 3 and 12 month creatinine) were compared.

**Results:**

Complete data was available on 129 paired kidneys.115 pairs were transplanted in the same centre and 14 pairs transplanted in different centres. There was a significant increase in CIT in kidneys transplanted second when both kidneys were accepted by the same centre (15.5 ± 4.1 vs 20.5 ± 5.8 hrs p < 0.0001 and at different centres (15.8 ± 5.3 vs. 25.2 ± 5.5 hrs p = 0.0008). DGF rates were increased in the second implant following sequential transplantation (p = 0.05).

**Conclusions:**

Paired study sequential transplantation of kidneys from DCD donors results in a significant increase in CIT for the second kidney, with an increased risk of DGF. Sequential transplantation from a DCD donor should be avoided either by the availability of resources to undertake simultaneous procedures or the allocation of kidneys to 2 separate centres.

## Background

In the United Kingdom (UK) kidneys from brain-dead donors (DBD) are allocated through a national organ sharing scheme that matches the donor to the best recipient.The UK Kidney Allocation Scheme introduced in April 2006 prioritises patients with ideal tissue matches (000 HLA mismatches) and blood group matching, then assigns points to patients based on the level of tissue match between donor and recipient, the length of time spent waiting for a transplant, age of the recipient (with a progressive reduction in points given after the age of thirty) and location points such that patients geographically close to the retrieval centre receive more points. The patients with the highest number of points for a donated kidney are preferentially offered the kidney, no matter where in the UK they receive their treatment.

In contrast, there is no standardized policy for allocation of kidneys retrieved from donors after circulatory death (DCD). Allocation is often based on regional and individual centre policy. The current practice in the Pan Thames region encompassing 5 units which serve the Greater London area and South Eastern England is a center based allocation wherein, a single centre is initially offered both kidneys from each donor on a rota basis. This centre usually transplants both kidneys although at times one kidney may be subsequently re-allocated to another centre. The implications of this allocation policy in terms of graft outcome have not been previously evaluated.

Whilst long term outcomes and graft survival are similar between DBDs and DCDs, it has been well established that kidneys from DCD’s have higher rates of primary non-function (PNF) and delayed graft function (DGF) than from DBD kidneys [1-4]. These differences impact on in-hospital stay and overall costs which are consequently much higher in DCD compared to DBD transplants. Primary warm ischemia time (WIT) and haemodynamic events around the retrieval period are likely to be responsible for the increase in DGF/PNF in DCD kidneys and is accepted as an inevitable consequence of use of this donor source [5]. DCD organs are being increasingly utilized due to the shortage of DBD kidneys and the ever expanding number of patients requiring a transplant. In the UK the use of kidneys from DCD donors has risen from 3% of all deceased donors in 2000 to 82% in 2012. Between April 2011 and March 2012, 674 kidney transplants from donors after circulatory death (17% increase compared with the previous year) took place and accounted for one in four of all kidney transplants [6]. Hence, it is important to identify and overcome factors which may adversely affect the outcome of DCD kidney transplants both in terms of patient interest and health care economics.

In addition to warm ischaemic damage, cold ischemia time (CIT), donor age, recipient body mass index and pre-transplant dialysis are factors contributing to the risk of DGF [7-9]. Of these, CIT is probably the most readily modifiable factor for both DBD and DCD transplantation [10-12]. Minimizing CIT reduces not only DGF but also acute rejection episodes and graft loss which are both increased in cases of DGF [11].

When both kidneys from a single donor are allocated to the same centre which has been the standard practice in our region, the recipient operations are most likely to occur sequentially rather than simultaneously due to staff and operating theatre availability. An inevitable anticipated consequence of this practice is a substantial difference in cold ischemia times between the two recipients which then could potentially impact on graft outcomes. Conversely, if kidneys from a given donor are sent to different centres’ in close proximity, then similar CIT’s for both organs should be the anticipated outcome.

In this audit we compared the outcomes between paired DCD kidneys within our regional allocation policy of both kidneys being offered to a single centre. Our aim was to determine whether this policy resulted in clinically significant delays in the implantation of the second kidney with the increased CIT impacting on DGF and patient outcome.

## Methods

Prospectively collected data on all adult recipients who had renal transplants from DCD’s within the Pan Thames region from April 2002 to March 2010 was reviewed. The Pan Thames region comprises five London transplant centres (Royal Free, Guy’s, West London, Royal London and St George’s) that provide renal transplantation to the Greater London and South Eastern England serving a population of approximately 12 million.

For the purposes of the audit we defined ‘simultaneous transplants’ as those occurring when there was <3 hours difference in CIT as this time difference is only feasible with access to 2 theatres. ‘Sequential transplants’ were defined as those with ≥3 hours difference in CIT between the two grafts. PNF was defined as a graft that never achieved sufficient function to allow discontinuation of dialysis and DGF as the need for dialysis in the first week after transplantation.

Donor details and recipient centres were identified from United Kingdom Transplant (UKT) registry. Recipient demographics, comorbidities, secondary warm ischaemic time, PNF, DGF, serum creatinine at three months and twelve months were retrieved from hospital records. Retrieval biopsies on donor kidneys are not performed routinely to evaluate the donors in the Pan Thames area. HLA-mismatching was not recorded in patient case notes.

Exclusion criteria included; un-linked data between the donor and recipient, missing data, kidneys exported outside the Pan Thames region and multi-organ transplantation (usually kidney-pancreas). All patients active on UK transplant list gave informed consent for data collection and analysis by UKT. Our audit was approved by the PanThames audit committee which has links to the PanThames health care commissioners. All chief executives of the hospitals involved in the audit were contacted and approval sought prior to the audit. This audit was conducted in accordance with the guidelines set out by the UK Department of Health.

### Statistical analysis

Analysis was done using STATVIEW (SAS Institute Inc., Cary, NC). Descriptive statistics was used to present demographic, transplant and outcome related data. Categorical variables were compared using Chi-square and Fisher’s exact test and continuous variables using unpaired‘t’ and Mann–Whitney ‘U’ test,. Logistic regression analysis was used to evaluate the impact of the order of implantation on DGF. Data is displayed as mean ± standard deviation, unless otherwise stipulated. Statistical significance was taken at the p < 0.05 level.

## Results

There were 326 DCD transplants during the study period. PNF occurred in 5 patients. A total of 63 donors were excluded because one of the paired kidneys was exported outside the region (15), or because incomplete data (48). Complete data was available on 129 paired kidneys. Both kidneys from each donor were initially offered to one of the 5 centres. A total of 115 pairs of kidneys were transplanted with both recipients at a single centre. In the case of the remaining 14 donors representing 11% of the total the kidneys were transplanted in 2 separate centres (Table 1).

**Table 1:** Allocation of paired kidneys from DCDs

**Allocation**	**Simultaneous implant-CIT < 3 hrs (n in pairs)**	**Sequential implant-CIT ≥3 hrs (n in pairs)**
Single centre: n = 115	24(21%)	91(79%)
2 Centre: n = 14	3(21%)	11(79%)
Total: N = 129	27(21%)	102(79%)

The demographics of the first and second recipients are shown in Table 2. These groups were comparable except for a higher proportion of patients on peritoneal dialysis in the first recipients.

**Table 2:** Differences between the first and second kidneys transplanted

		**Same centre transplant**			**2 centre transplant**			**Total transplants**		
		**Kidney 1**^**st**^	**Kidney 2**^**nd**^	**p-value**	**Kidney 1**^**st**^	**Kidney 2**^**nd**^	**p-value**	**Kidney 1**^**st**^	**Kidney 2**^**nd**^	**p-value**
		**n = 115(%)**	**n = 115(%)**		**n = 14(%)**	**n = 14(%)**		**n = 129(%)**	**n = 129(%)**	
Recipient gender	Male	78(68)	77(67)		8(57)	8(57)		86(67)	85(66)	
	Female	37(32)	38(33)		6(43)	6(43)		43(33)	44(34)	
Dialysis	Pre-	6(5)	4(3)		2(14)	3(21.5)		8(6)	7(5)	
	PD	44(38)	25(22)	0.0004	6(43)	3(21.5)		50(39)	28(22)	0.007
	HD	65(57)	86(75)		6(43)	8(57)		71(55)	94(73)	
Recipient HTN	Yes	48(42)	45(39)		2(14)	8(57)	0.046	50(39)	53(41)	
	No	67(58)	70(61)		12(86)	6(43)		79(61)	76(59)	
Recipient Diabetes	Yes	18(16)	10(9)		2(14)	3(22)		20(16)	13(10)	
	No	97(84)	105(91)		12(86)	11(78)		109(84)	116(90)	
Recipient IHD	Yes	11(10)	6(5)		1(7)	1(7)		12(9)	7(5)	
	No	104(90)	109(95)		13(93)	13(93)		117(91)	122(95)	
DGF	Yes	64(56)	77(67)		8(57)	7(50)		72(56)	84(65)	
	No	51(44)	38(33)		6(43)	7(50)		57(44)	45(35)	
Recipient age (Years ± SD)		50.5 ± 12.6	49.3 ± 12.8		49.4 ± 14.8	48.7 ± 10.2		50.4 ± 12.8	49.2 ± 12.5	
CIT (hours ± SD)		15.5 ± 4.1	20.5 ± 5.8	<0.0001	15.8 ± 5.3	25.2 ± 5.5	0.0008	15.5 ± 4.2	20.9 ± 5.9	<0.0001
Sec. warm ischaemia time (min ± SD)		38.0 ± 13.8 (n = 85)	34.1 ± 11.8 (n = 85)	0.047	40.5 ± 7.4	34.5 ± 12.7				
Creatinine at 3 mths (umols/L ± SD)		186.6 ± 173.1	177.7 ± 125.1		140.4 ± 49.4	141.6 ± 45.4		181.8 ± 165.2	170.7 ± 123.1	
Creatinine at 12 months (umols/L ± SD)		168.8 ± 155.7	169.6 ± 173.4		122.5 ± 24.2	136.4 ± 43.5		164.1 ± 148.3	165.7 ± 163.9	

Comparison between the 1^st^ and 2^nd^ Kidney: All values of p = NS unless otherwise stated.

### Cold ischaemia times

#### Paired kidneys same centre transplant

In cases where both kidneys from the same donor were transplanted in a single centre there was a significant increase in CIT for the second kidney (15.5 ± 4.1 vs. 20.5 ± 5.8 hrs p < 0.0001). This represented a mean and median difference in CIT between paired kidneys of 4.9 ± 3.2 hours (±SD) and 4.0 hours respectively (IQR 3) (Table 3). The secondary WIT was also significantly shorter in the kidneys transplanted second although complete data was available only in 85 paired kidneys (38.0 ± 13.8 vs. 34.1 ± 11.8 minutes p = 0.047) (Table 2).

**Table 3:** Cold ischemia times of DCD kidney pairs transplanted at single/multiple centres

		**Kidney 1**	**Kidney 2**	**p-value**
Single centre	Mean (hrs ± SD)	15.5 ± 4.1	20.5 ± 5.8	<0.0001
	Median [hrs(IQR)]	15( 5.2)	19.25( 5.9)	
Two centres	Mean (hrs ± SD)	15.8 ± 5.3	25.2 ± 5.5	0.0008
	Median [hrs(IQR)]	16.5( 8.6)	24(5.5)	
Total	Mean (hrs ± SD)	15.5 ± 4.2	20.9 ± 5.9	<0.0001
	Median [hrs(IQR)]	15.3( 5.5)	19.5( 6.3)	

#### Paired kidneys different centre transplant

The CIT in kidneys transplanted second was also significantly increased when the kidneys were used by different centres (15.8 ± 5.3 vs. 25.2 ± 5.5 hours p = 0.0008) with the mean and median difference in CIT between kidneys of 9.5 ± 6.5 hrs (±SD) and 9.0 hrs respectively (Table 3).

Review of patient details for these transplants indicated that this delay related to unsuitability of a planned recipient at the first centre due to either a positive cross match or recipient medical issues resulting in secondary reallocation. Delays consequent to this arose due to the need for further organ transport, recipient identification, and crossmatching at the second centre.

#### Second kidneys only–single versus different centre

The CIT of the second kidney was significantly longer when this was transplanted at a second centre compared to when both kidneys were used at the same centre (20.5 ± 5.8 vs. 25.2 ± 5.5 hours, p = 0.01). The number of simultaneous transplants was identical with single centre and 2 centre recipient operations comprising 21% (24/115 and 3/14) for both scenarios.

#### Short-term graft function (Serum creatinine levels)

The mean serum creatinine levels at 3 months for the first and second kidneys implanted with CIT of 3 hours difference from the same donor were 161.3 and 159.1 umol/l respectively. At 12 months it was 174.0 and 154.0 umol/l. However, it did not achieve statistical significance for the transplants performed with more than 4 hours of CIT difference (p = 0.55).

### Delayed graft function

A total of 157/258 kidneys had primary function and 101/258 developed DGF. The median length of stay for the DGF group was 12 days. Regression analysis did not show any correlation between the number of days of DGF and serum creatinine at either 3 or 12 months (p = 0.09 & 0.25 respectively).

There was a trend towards increased incidence of DGF in those kidneys transplanted second although it did not achieve statistical significance (p = 0.078). In addition, there was no significant difference in any of the recipient factors or short-term outcomes in terms of 3 and 12 month serum creatinine between the two groups (Table 2).

However when we analysed paired kidneys with CIT difference ≥3 hours showed an increased incidence of DGF in the kidneys transplanted second [p = 0.05, RR 1.82 (95% CI 1–3.32)]. The difference in DGF between first and second kidneys was further increased when the difference in CIT was >4 hours [p = 0.01, RR 2.6 (95% CI 1.3-5.3)]. In this cohort, despite the significantly higher peritoneal dialysis (PD) rate among the first kidney transplant group (p = 0.006), the mode of dialysis (PD vs. Haemodialysis) did not influence the incidence of DGF (p = 0.64). However DGF was lower in patients who were pre-emptive versus those being on either mode of dialysis (p = 0.016, RR 13.2, 95% CI 1.6-107.7). These patients were equally distributed between first and second kidney groups (Table 2). The order of implantation continued to significantly influence the incidence of DGF after controlling for pretransplant dialysis (Table 4). Pre transplant dialysis did not influence the incidence of DGF in this cohort (p = 0.06).

**Table 4:** Difference in DGF between the first and second kidneys transplanted at the same centre- subgroups based on differences in CIT

**Difference in CIT**	**Incidence of DGF**		**Odds ratio (95% CI)**	**Odds ratio (95% CI)**
	**1**^**st **^**kidney**	**2**^**nd **^**kidney**		**Controlled for pre-transplant dialysis**
<3 hours	67% (16/24)	67% (16/24)	NS	-
≥3 hours	53% (48/91)	67% (61/91)	1.8 (1–3.32) p = 0.05	1.9 (1–3.5) p = 0.046
≥4 hours	48% (31/64)	70% (45/64)	2.6 (1.3-5.3) p = 0.01	2.7 (1.3-5.6) p = 0.009

## Discussion

This study reviews the outcome of a local allocation policy for DCD kidneys and the consequent impacts on CIT and graft function. In the Pan Thames region both kidneys from individual DCD donors have been primarily allocated to one of 5 centres on a rota basis. With this mechanism of allocation and utilization there was a substantial effect on CIT disadvantaging the second kidney from most donor pairs. Approximately 80% of kidneys were transplanted sequentially.

When both kidneys were used at a single centre, which occurred in 90% of cases, the mean difference in CIT between the first and second implants approached 5 hours. This is highly likely to reflect logistic issues related to theatre and team constraints. Fixed delays contributing to CIT including transport of organs and blood, cross matching and patient preparation generally would equally apply to both recipients. National UK data has demonstrated an adverse effect of CIT on DGF and outcome when this exceeds 12 hours [10]. Based on our experience in the Pan Thames region, this target was generally not met with current clinical practice even with the first of most pairs of kidneys. Whilst various strategies, including virtual cross matching, have been introduced to reduce CIT, resource limitations at individual units mostly restrict achieving this target for both kidneys from a single donor. The present allocation policy thus inherently compromises a substantial number of recipients of DCD kidneys.

An important observation is that where simultaneous transplantation of both organs was achieved, based on differences in CIT of <3 hours, the incidence of DGF was identical with both kidneys. This scenario, however, was only achieved in 20% of cases. With differences beyond 3 hours, which occurred in the majority of cases, there was a negative effect on DGF with increasing time to transplantation in the second kidney. This is consistent with previous publications [4].

Patients on dialysis are known to be at increased risk of DGF following cadaveric renal transplantation as compared to those undergoing pre-emptive transplantation. Additionally, the type of dialysis might also influence DGF in such patients [13]. In our study we observed a significant difference in the mode of dialysis between first and second graft recipients (Table 2). In single centre transplants there were a higher proportion of patients undergoing peritoneal dialysis in the recipients of the first kidney compared to the second kidney. Although there was no difference in rates of DGF between the different modalities of dialysis, there was a significant difference in the rate of DGF between patients on dialysis and those having pre-emptive transplants. Multiple logistic regression confirmed that CIT difference of ≥ 3 hours continued to remain an independent risk factor for DGF irrespective of pre-transplant dialysis status.

One potential mechanism to minimize the CIT of the second kidney from a DCD donor would be the allocation to 2 separate institutions. This only occurred in a minority of cases and again generally resulted in sequential transplantation. Unfortunately of great concern was the finding that paradoxically this had an even greater adverse effect on the CIT of the second kidney. Examining individual cases this reflected the initial acceptance of both kidneys by one institution followed by a secondary allocation occurring when the first centre was unable to proceed with both transplants. Various factors contributed to this, including positive crossmatch and recipient medical unsuitability without the ready availability of additional suitable recipients, as well as technical concerns regarding one of the kidneys by the surgical team at the initial centre. As a consequence of this process which required subsequent recipient identification, organ transport and cross matching very substantial delays occur evidenced by the prolonged CIT of approximately 10 hours for the second kidney.

The secondary WIT was found to be significantly lower for the kidney transplanted second at the same centre. However the time difference noted is unlikely to be of clinical consequence and would not obviously account for any of the outcomes noted.

Organ allocation from deceased donors can be both controversial and dictated by local and regional factors including resources as well as the recipient population. This is compounded by the shortage of organs relative to the number of patients who would benefit from renal transplantation. There is substantial variation between and within individual countries. Algorithms guiding allocation need to incorporate equity of access for potential recipients balanced by the desire to ensure the best possible outcomes.

Health care costs vary between institutions and from country to country. The current system in the London region in the UK may address equity of organ allocation, although we have not specifically examined this. It, however, does not ensure the optimal outcomes for all kidneys based on this paired study. Infrastructure limitations within transplant centres that prevent simultaneous transplantation of both organs may compromise optimal outcomes.

The current accommodation cost for one day in our renal transplant ward is £207 ($326) and our median length of in-patient stay for renal transplants with DGF is 12 days, significantly greater than 6 days for those transplants with primary function. Thus DGF adds a minimum additional £1242 ($1956) to the cost of the transplant just for the increased hospital bed stay. However this excludes the cost of medicines (immunosuppressants), additional blood tests and other investigations, including renal transplant biopsy, as our standard practice is to biopsy all transplants with DGF at day 7.

We propose that kidneys from DCD donors should be allocated through a mechanism to maximize the possibility of simultaneous transplantation to ensure prolongation of the CIT of the second kidney does not occur. This should be considered on a regional rather than national level to minimize overall CIT.

We would recommend that for renal transplantation using DCD allografts that if both kidneys are sent to the same centre two contemporaneous transplants in separate operating theatres should be performed to minimize the risk of DGF and longer term renal injury [14]. In our opinion, allocation of kidneys from each DCD donor should be to 2 institutions within close geographical proximity.

Whilst some institutions may at times be able to accommodate 2 transplants simultaneously, our study suggests that in practice this is generally unlikely in most transplant centres. Institutions accepting these kidneys should ensure a backup recipient is available to avoid the current practice of a secondary allocation process, which has been shown to result in a profound and unacceptable delay in the majority of cases in which this occured. A workable arrangement incorporating these requirements may be close cooperation between collaborating units to provide a functional alternative to the current arrangement.

A pilot collaboration programme has been established between 2 transplant centres (Royal Free & Royal London Hospitals) where they share the 2 kidneys from a single DCD donor if offered to one of the two units. This has helped to minimize most of the practical and logistical problems of performing simultaneous transplants in the same center hence with a shorter CIT. Given the initial promising positive results of this shared acceptance system of 2 kidneys, then it could be applied to other neighbouring transplant units in greater London.

As a method of increasing the organ utilization we have expanded the acceptance criteria for marginal donors, so that in our region we transplant two marginal DCD kidneys into a single recipient (dual renal transplant). The early outcomes data showed that transplanting two marginal kidneys, otherwise destined to be discarded is an appropriate option for selected recipients. However if we were to offer dual transplantation of all DCD kidneys, then this would halve the number of potential recipients.

## Conclusion

The current primary allocation of both kidneys from a DCD donor to single institutions within a UK transplant region exposes the second kidney of the majority of pairs to a significantly higher CIT and consequent risk of DGF. Based on our experience, sequential transplantation results in unacceptable CIT for the second kidney transplanted from a DCD donor. Hence, it would be advisable to share the paired kidneys based on a regional system involving units in close proximity or if offered to the same centre, ensure logistical support for simultaneous transplantation. Additionally, following initial acceptance by a centre, secondary re-allocation leads to an unacceptable prolongation of CIT in the majority of cases. Where possible this should be avoided with back up patients available to ensure that the unsuitability of the initial planned recipient does not adversely affect the CIT of the kidney.

We would also recommend other regions with similar allocation protocols (i.e. both organs from a DCD donor allocated to the same centre) review their performance and outcomes, particularly with respect to the second kidney of each pair. It is likely that the issues and outcomes we have highlighted are widespread. This may lead to a formal review of DCD kidney allocation across the UK and other countries to improve the overall outcomes from DCD donor kidney transplants.

## Abbreviations

DCD: Donation after circulatory death; CIT: Cold ischemia time; DGF: Delayed graft function; PNF: Primary non-function; WIT: Warm ischemia time; UKT: United Kingdom transplant; DBD: Donation after brain death.

## Competing interests

The authors of this manuscript have no competing interests to declare.

## Authors’ contributions

SB- was responsible for the design and conduct of the project, interpretation of results, and composition of all sections of the manuscript. TT carried out data collection for all the donors, BJ did the statistical analysis, BL helped out with data analysis, DN participated in the designing & initiation of the study, JO, MD, NM, FC, NK, JT, GK, MM, RC, CP, SD, AS, NH, VP- carried out data collection & analysis of the data, AD helped to obtain approval for the audit from the PanThames audit committee and helped draft the manuscript & corrections, BF supervised the whole study & as the senior author. All authors read and approved the final manuscript.

## Pre-publication history

The pre-publication history for this paper can be accessed here:

http://www.biomedcentral.com/1471-2369/15/83/prepub
